# Unmatched ventilation and perfusion measured by electrical impedance tomography predicts the outcome of ARDS

**DOI:** 10.1186/s13054-021-03615-4

**Published:** 2021-06-03

**Authors:** Elena Spinelli, Michael Kircher, Birgit Stender, Irene Ottaviani, Maria C. Basile, Ines Marongiu, Giulia Colussi, Giacomo Grasselli, Antonio Pesenti, Tommaso Mauri

**Affiliations:** 1 Department of Anesthesia, Critical Care and Emergency, Fondazione IRCCS Ca’ Granda Ospedale Maggiore Policlinico, Via Francesco Sforza 35, 20122 Milan, Italy; 2 Institute of Biomedical Engineering, Karlsruhe Institute of Technology, Karlsruhe, Germany; 3 Drägerwerk AG & Co. KGaA, Lübeck, Germany; 4 Department of Pathophysiology and Transplantation, University of Milan, Milan, Italy

**Keywords:** Acute respiratory distress syndrome, Electrical impedance tomography, Positive end expiratory pressure, Ventilation/perfusion matching

## Abstract

**Background:**

In acute respiratory distress syndrome (ARDS), non-ventilated perfused regions coexist with non-perfused ventilated regions within lungs. The number of unmatched regions might reflect ARDS severity and affect the risk of ventilation-induced lung injury. Despite pathophysiological relevance, unmatched ventilation and perfusion are not routinely assessed at the bedside. The aims of this study were to quantify unmatched ventilation and perfusion at the bedside by electrical impedance tomography (EIT) investigating their association with mortality in patients with ARDS and to explore the effects of positive end-expiratory pressure (PEEP) on unmatched ventilation and perfusion in subgroups of patients with different ARDS severity based on PaO_2_/FiO_2_ and compliance.

**Methods:**

Prospective observational study in 50 patients with mild (36%), moderate (46%), and severe (18%) ARDS under clinical ventilation settings. EIT was applied to measure the regional distribution of ventilation and perfusion using central venous bolus of saline 5% during end-inspiratory pause. We defined unmatched units as the percentage of only ventilated units plus the percentage of only perfused units.

**Results:**

Percentage of unmatched units was significantly higher in non-survivors compared to survivors (32[27–47]% vs. 21[17–27]%, *p* < 0.001). Percentage of unmatched units was an independent predictor of mortality (OR 1.22, 95% CI 1.07–1.39, *p* = 0.004) with an area under the ROC curve of 0.88 (95% CI 0.79–0.97, *p* < 0.001). The percentage of ventilation to the ventral region of the lung was higher than the percentage of ventilation to the dorsal region (32 [27–38]% vs. 18 [13–21]%, *p* < 0.001), while the opposite was true for perfusion (28 [22–38]% vs. 36 [32–44]%, *p* < 0.001).

Higher percentage of only perfused units was correlated with lower dorsal ventilation (*r* =  − 0.486, *p* < 0.001) and with lower PaO_2_/FiO_2_ ratio (*r* =  − 0.293, *p* = 0.039).

**Conclusions:**

EIT allows bedside assessment of unmatched ventilation and perfusion in mechanically ventilated patients with ARDS. Measurement of unmatched units could identify patients at higher risk of death and could guide personalized treatment.

## Background

Acute respiratory distress syndrome (ARDS) is characterized by diffuse bilateral inflammatory lung edema: neutrophils migrate into the alveoli and cytokines are released, compromising the anatomical integrity of the lung parenchyma and its functions [1]. Such alterations profoundly impact the physiological distribution of ventilation and perfusion. Fluid extravasation fills the interstitium and the alveoli and increases lung weight, determining regional de-recruitment and hypoventilation [2]. Airways resistance can be increased regionally by airway closure [3], surfactant depletion [4] and alveolar hypocapnia[5], further diverting ventilation. Perfusion becomes more heterogeneous due to hypoxic vasoconstriction [6], diffuse micro-thrombosis[7, 8] and compression [9]. As a result, perfused regions that are non-ventilated coexist with ventilated regions that are non-perfused, and their amount should mirror the intensity of the processes determining ARDS severity. Larger areas of unmatched ventilation and perfusion might also increase the risk of ventilation-induced lung injury (VILI), because of the higher pressure needed to recruit and keep open the collapsed lung [10], and of the higher minute ventilation required in the presence of elevated dead space [11]. In summary, the extent of unmatched ventilation and perfusion might be correlate with ARDS outcome through a mix of higher baseline severity plus higher risk of VILI.

Electrical impedance tomography (EIT) is a dynamic bedside monitor of lung volume changes [12], able to accurately assess regional ventilation distribution in ARDS patients [13, 14]. EIT-based method that quantitatively estimates regional lung perfusion based on first-pass kinetics of a bolus of hypertonic saline contrast has been developed [15], validated in experimental studies [16–19], and recently increasingly applied in critically ill patients [20–24]. EIT might identify only ventilated (i.e., ventilated but not perfused) units and only perfused (i.e., perfused non-ventilated) units expressed as a percentage of total lung volume. The sum of these two types of unmatched units is an assessment of the extent of ventilation/perfusion unmatching in each patient.

In the present study, we reasoned that EIT-based measurement of unmatched units might improve our ability to characterize ARDS severity at the bedside. Thus, we prospectively assessed unmatched units in a relatively large cohort of intubated patients with ARDS, early in their clinical course. The study hypothesis was that higher percentage of unmatched units might be correlated with poorer clinical outcomes. Moreover, we explored the correlations between unmatched units and ARDS pathophysiology and the effect of positive end expiratory pressure (PEEP) in subgroups of patients with different ARDS severity.

## Methods

We prospectively enrolled 50 patients admitted to the Intensive Care Unit (ICU) of the Ospedale Maggiore Policlinico (Milan, Italy). Inclusion criteria were: fulfilment of the diagnostic criteria for ARDS according to the Berlin definition [25]; intubation, deep sedation and paralysis with controlled mechanical ventilation; central venous catheter in place. Exclusion criteria: age < 18 years; hemodynamic instability (i.e., severe hypotension with systolic arterial pressure < 60 mmHg despite fluid expansion and vasoactive support; systolic arterial pressure > 180 mmHg; hemodynamically significant cardiac arrhythmias); pregnancy; contraindications to the use of EIT (e.g., presence of pacemaker or chest surgical wounds dressing). The study was approved by the Ethical Committee of the Policlinico Hospital (reference number 513_2019) and informed consent was obtained according to local regulations.

### Study protocol and EIT measurements

**Fig. 1:**
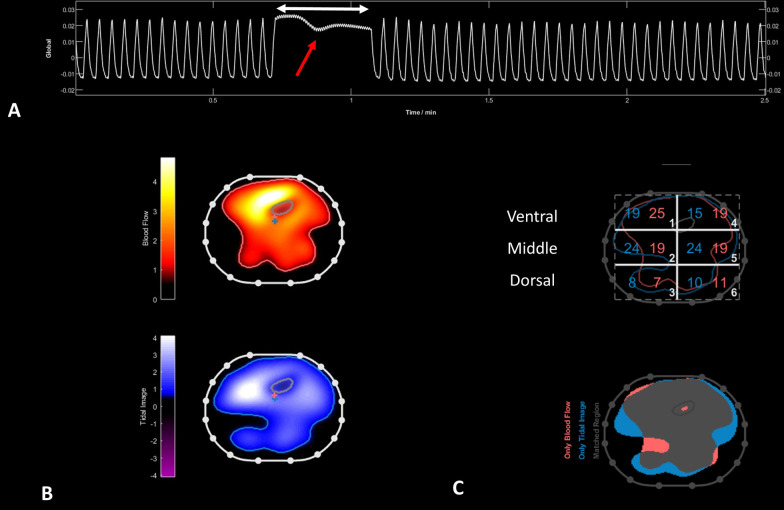
Ventilation and perfusion measured by EIT in a representative patient. **A** Electrical impedance tomography signals during the infusion of saline bolus. The white arrow indicates the duration of the end-inspiratory hold during which the saline bolus is administered. Note that changes in impedance due to tidal ventilation are interrupted; the drop in impedance due to the bolus pass is indicated by the red arrow. **b** Representative image of the perfusion (red-colour map) and ventilation (blue-colour map) distribution. Note the gray circle indicating the cardiac region that has been removed from the analysis. **c** Top: representative map with the percentage perfusion (red numbers) and ventilation (blue numbers) in the three lung regions. Bottom: representative map obtained by integrating ventilation and perfusion maps: the grey area indicates matched units which are both ventilated and perfused, while red area indicated only perfused units and blue area only ventilated units

Ventilation mode, tidal volume (Vt), respiratory rate (RR), PEEP, and fraction of inspired oxygen (FiO_2_) were left as selected by the attending physician throughout the whole study. Patients were kept in the semi-recumbent position. An EIT dedicated belt with 16 electrodes was placed around the patient's chest at the fifth or sixth intercostal space and connected to an EIT monitor (PulmoVista® 500, Dräger, Lübeck, Germany). EIT data were acquired at a frame rate of 50 Hz by injecting small electrical currents at adjacent electrode pairs around the patient’s thorax and stored for offline analysis After a baseline recording of EIT data for 5 min, we performed an end-inspiratory breath hold lasting 20 s. After 2 s from start of the occlusion, a bolus of 10 ml of 5% NaCl solution was manually injected via the central venous catheter. The bolus of saline solution, injected in less than 2 s, passes through the pulmonary circulation producing an impedance dilution curve that follows typical first-pass kinetics [15] (Fig. 1a).

The EIT signals were recorded at a frame rate of 50 Hz. *EIT ventilation maps* (Fig. 1b, bottom) were obtained from offline analysis of tracings by averaging values over five consecutive respiratory cycles. We horizontally split the EIT images into three contiguous regions of interests of the same size: ventral, middle, and dorsal. From the analysis of ventilation maps we measured:

- the pixel-level ventilation measured as impedance change between expiration and inspiration. Pixels were then classified as non-ventilated if pixel ventilation was ≤ 10% of the highest pixel-level value measured in that patient
- the percentage of ventilated pixels in the respective region (ventral, middle and dorsal) (Fig. 1c, blue)

*EIT perfusion maps* (Fig. 1b, top) were derived from offline analysis of the time-impedance curve obtained during the first pass of the saline solution during occlusion, after removing the cardiac region from the images. Briefly, a ventricular region was detected combining information from both pulsatility and indicator dilution-based EIT signal components in order to increase the robustness of the approach. Pulsatility signals were computed based on high-pass filtering, pulse detection and subsequent ensemble averaging. For the image entries a similarity measure of the respective pulsatility signal was evaluated mutually in combination with criteria regarding signal power and statistical information on shape and location of the region of interest in humans. An indicator-based heart region was determined by K-Means clustering using three features of the indicator signal, as previously described[19]. The underlying assumption is that three functional compartments are involved: cardiac chambers and large vessels upstream of the lung, the lungs as second compartment and a compartment downstream of the lungs. The final ventricular region was formed by the conjunction of the results from pulsatility and indicator-based measurements.

From the analysis of perfusion maps we measured:

- the relative pixel-level perfusion [15, 17]: after preprocessing, the steepest (maximal) slope of the temporal EIT signal deflection during the saline bolus injection in each pixel was normalized to the overall detected signal, yielding the relative pixel perfusion. Pixels were classified as non-perfused if pixel perfusion was ≤ 10% of the highest pixel-level value measured in that patient
- the percentage of perfusion in each region (ventral, middle, and dorsal) (Fig. 1c, red)

By integrating the pixel-level data on ventilation and perfusion (Fig. 1c, bottom), we calculated:

- % of only perfused units: corresponding to the non-ventilated pixels divided by the total number of pixels classified as ventilated and/or perfused
- % of only ventilated units: corresponding to the non-perfused pixels divided by the total number of pixels ventilated and/or perfused
- % of unmatched units: computed as the sum of only perfused plus only ventilated units
- Only ventilated/only perfused units ratio: calculated as the ratio between the percentage of only ventilated and the percentage of only perfused units
- Relative ventilation/perfusion ratio in each region: corresponding to the percentage of ventilation divided by the percentage of perfusion in each region

### Data collection

At enrollment, the following data were collected: demographic data, ARDS etiology, Simplified Acute Physiology Score II (SAPS II) at ICU admission, controlled mechanical ventilation settings and arterial blood gas analysis. End-inspiratory and end-expiratory holds were performed to collect total PEEP, plateau pressure, driving pressure, and compliance of the respiratory system. The ventilatory ratio was calculated as previously described[26]. Outcome data collected after the end of the protocol included: hospital mortality and ventilator-free days (VFDs) at day 28.

### Study endpoints

The aims of the study were:

1. To investigate whether higher percentage of unmatched units measured by EIT predicts mortality
2. To explore the regional distribution of ventilation/perfusion (V/Q) ratio and correlations between unmatched units and ARDS pathophysiology
3. To explore the effect of PEEP on the percentage unmatched units and on the ratio between only ventilated and only perfused units in subgroups of patients with different ARDS severity based on PaO_2_/FiO_2_ and compliance of the respiratory system (lower and higher than the median value of the study population).

### Statistical analysis

To calculate study sample size, we hypothesized that a 10% difference in percentage of unmatched units might be clinically relevant and identify patients with higher risk of death. By assuming an overall hospital mortality of 30% for the study population and 20 ± 9% of unmatched units in survivors, a sample of 46 patients was required to detect a difference of ≥ 10% in V/Q uncoupling between survivors and non-survivors with a power of 0.8 and alpha 0.05. We decided to include 50 patients to compensate for potential dropouts. Comparisons between target physiological variables in patient subgroups were performed by t test or by Wilcoxon signed rank test, as appropriate. Multivariate analysis was performed for prediction of clinical outcomes (i.e., mortality and ventilator-free days a day 28) based on a backward stepwise selection algorithm. In brief, all EIT-derived measurements (% of only ventilated units, percentage of unmatched units and only ventilated/only perfused units ratio) associated with outcome at univariate analysis (*p* < 0.05) were inserted in the multivariate analysis, while adjusting for clinical severity (SAPS II and SOFA scores). Odds ratios (OR) for mortality risk were provided with 95% confidence interval (CI).

The discriminative performance of the identified independent predictor of mortality was further evaluated by constructing receiver operating characteristics (ROC) curves and Youden Index was used to identify the optimal cutoff value. Correlation between continuous variables was assessed by Spearman regression coefficient. Normally distributed data are indicated as mean ± standard deviation, while median and interquartile range [IQR] are used to report non-normally distributed variables. Normality was tested by the Shapiro–Wilk test. A level of *p* < 0.05 (two-tailed) was considered as statistically significant. Statistical analyses were performed by SigmaPlot 11.0 (Systat Software Inc., San Jose, CA).

## Results

### Patient population

Patients main characteristics are listed in Table 1. Nine patients (18%) had severe ARDS (PaO_2_/FiO_2_ ≤ 100 mmHg and/or undergoing veno-venous extra-corporeal membrane oxygenation), 23 (46%) had moderate ARDS (PaO_2_/FiO_2_ > 100 mmHg and ≤ 200 mmHg) , and 18 (36%) had mild ARDS (PaO_2_/FiO_2_ > 200 mmHg and ≤ 300 mmHg). Median PEEP was 11 [8–15] cmH_2_O. Mean values of driving pressure and plateau pressure were 11 ± 4 cmH_2_O and 22 ± 4 cmH_2_O, respectively. Days between intubation and enrollment in the study were 2 ± 4 (i.e., early ARDS) and hospital mortality was 26% (13 out of 50 patients). Ten patients (20%) underwent prone positioning before the study.

**Table 1:** Patients’ main characteristics

Age, years	58 ± 15
Female, no. (%)	12 (24)
BMI, Kg/m^2^	26.5 ± 4.6
SAPSII at ICU admission	42 ± 13
Infectious/non-infectious ARDS, no. (%)	35 (70)/15 (30)
Pulmonary/extrapulmonary ARDS, no. (%)	24 (48)/26 (52)
PaO_2_/FiO_2_, mmHg	168 [122–236]
*Mechanical ventilation settings*	
PEEP, cmH_2_O Vt, ml/kg PBW Driving pressure, cmH_2_O Plateau pressure, cmH_2_O Respiratory rate, bpm	11 [8–15] 6.9 ± 1.7 11 [8–14] 23 [19–26] 16 [14–22]
Days of IMV before the study day, no	2 ± 4
Non-survivors, no. (%)	13 (26%)

BMI, body mass index; SAPSII, Simplified Acute Physiology Score II; PaO_2_, arterial oxygen tension; FiO_2_, fraction of inspired oxygen; PEEP, positive end-expiratory pressure; Vt, tidal volume; PBW, predicted body weight; IMV, invasive mechanical ventilation

### EIT-based measurements and clinical outcomes

Clinical severity, ventilation settings, and the EIT-based ventilation and perfusion parameters in the whole study population, and in survivors compared to non-survivors are showed in Table 2.

**Table 2:** Differences between survivors and non-survivors

	All (*n* = 50)	Survivors (*n* = 37)	Non-survivors (*n* = 13)	*P*-values Survivors versus non-survivors
Age, years	60 [46–69]	59 [42–70]	60 [48–71]	0.472
SAPSII at ICU admission	42 ± 13	38 ± 11	52 ± 14	< 0.001
PEEP, cmH_2_O	11 [8–15]	11 [8–15]	12 [8–13]	0.664
Vt, ml/Kg PBW	6.9 ± 1.7	6.8 ± 1.8	7.5 ± 0.9	0.184
Respiratory rate	16 [14–22]	16 [8–21]	16 [16–22]	0.128
Driving pressure cmH_2_O	11 ± 4	10 ± 3	12 ± 4	0.076
Plateau pressure, cmH_2_O	22 ± 4	22 ± 4	23 ± 4	0.493
Respiratory system compliance, ml/cmH_2_O	41 [35–51]	41 [35–52]	44 [33–52]	1.0
PaO_2_/FiO_2_, mmHg	168 [122–236]	169 [125–227]	166 [110–266]	0.707
Ventilatory ratio	1.4 [0.9–1.9]	1.5 [1.2–2]	1.4 [1.2–1.9]	0.596
Ventral ventilation, %	32 [27–38]	32 [26–38]	35 [24–42]	0.445
Ventral perfusion, %	28 [22–37]	27 [21–36]	29 [24–40]	0.528
Middle ventilation, %	51 [44–56]	50 [44–57]	51 [48–55]	0.956
Middle perfusion, %	55 [49–57]	55 [50–58]	50 [46–58]	0.665
Dorsal ventilation, %	18 [13–21]	19 [14–21]	13 [9–22]	0.126
Dorsal perfusion, %	36 [32–44]	35 [32–44]	40 [31–46]	0.618
Unmatched units, %	25 [18–31]	21 [17–27]	32 [27–47]	< 0.001
Only-perfused units, %	10 [5–16]	8 [5–11]	19 [11–35]	0.002
Only ventilated units, %	12 [9–18]	12 [9–18]	14 [7–22]	0.485
Only ventilated/only perfused units ratio	1.6 [0.7–2.4]	1.7 [0.8–2.6]	0.6 [0.3–2.2]	0.040

SAPSII, Simplified Acute Physiology Score II; SOFA, Sequential Organ Failure Assessment; PEEP, positive end-expiratory pressure; FiO_2_, fraction of inspired oxygen; Vt, tidal volume; PBW, predicted body weight; PaO_2_, arterial oxygen tension; V/Q, ventilation/perfusion

**Fig. 2:**
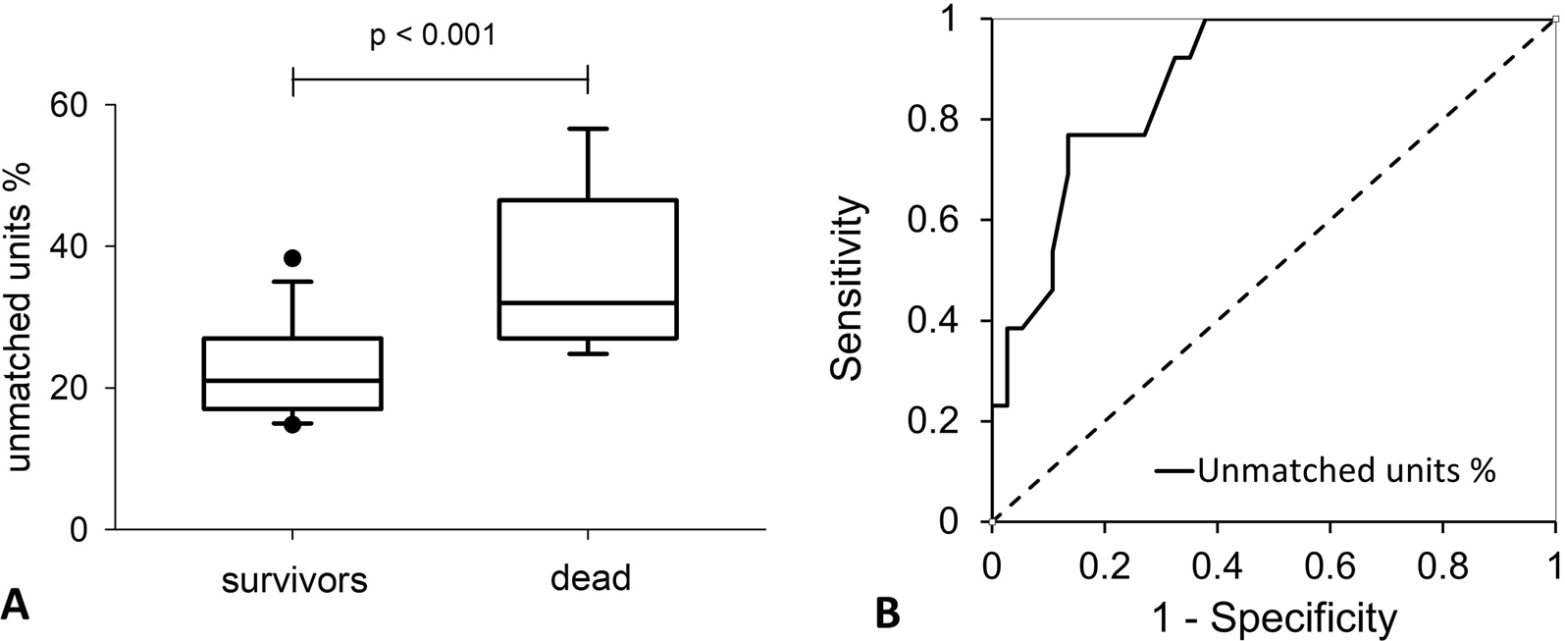
Representative EIT images. **a** Patient with 15% of unmatched units. **b** Patient with 57% of unmatched units

As expected, non-survivors had higher SAPS II at ICU admission (*p* < 0.001). Instead, driving pressure, compliance of the respiratory system, PaO_2_/FiO_2_ ratio and the ventilatory ratio (i.e., the efficiency in CO_2_ clearance)[26] were not different between survivors and non-survivors. Non-survivors presented higher percentage of unmatched units (Fig. 2a), higher percentage of only perfused units and lower only ventilated/only perfused units ratio (Table 2).

The adjusted multivariate logistic regression analysis showed that higher percentage of unmatched units was the only independent risk factor for mortality (OR 1.22, 95% CI 1.07–1.39, *p* = 0.004) after adjustment for clinical severity (SAPS II and SOFA score). Area under the ROC curve was 0.88 for percentage of unmatched units (95% CI 0.79–0.97, *p* < 0.001) (Fig. 2b). A cutoff value of 27% for unmatched units predicted mortality with a sensitivity of 77%, a specificity of 87%, a positive predictive value of 67% and a negative predictive value of 91%.

**Fig. 3:**
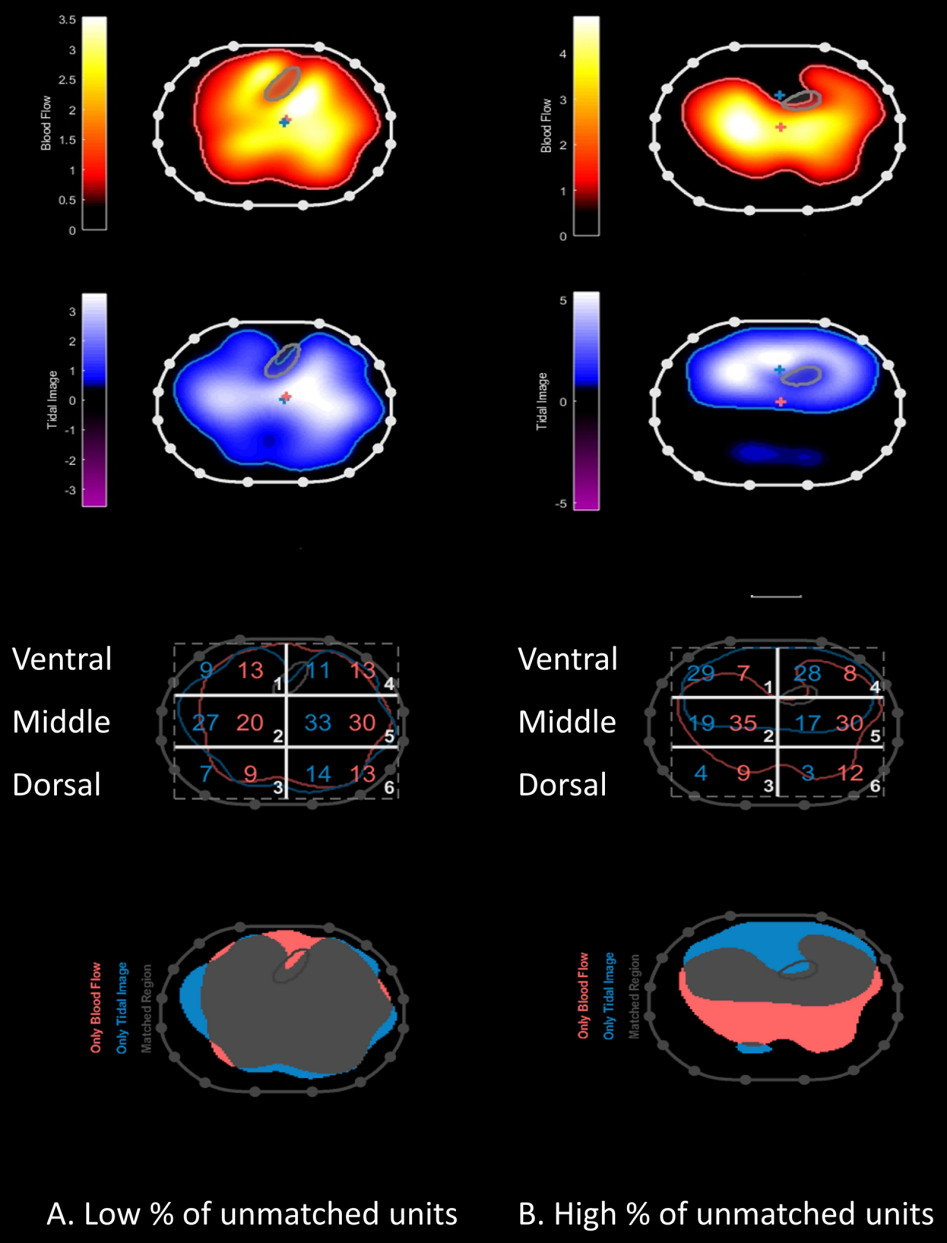
Percentage of unmatched units and clinical outcome. **a** Unmatched units in survivors versus non-survivors. For each boxplot, the line within the box indicate the median value, the box boundaries indicate 25th and 75th percentiles, and the error bars indicate the 5th and 95th percentiles. *P*-values for Wilcoxon-signed rank test. **b** ROC curve for SAPS II and unmatched units. **c** Correlation between unmatched units and ventilator-free days

Higher percentage of unmatched units was also the only independent predictor of less VFDs at day 28 (*r* =  − 0.415, *p* = 0.004) in the whole population, after adjusting for clinical severity (SAPS II and SOFA score). When the same analysis was performed only in survivors, the correlation was lost. Figure 3 shows representative images and maps of ventilation and perfusion in two patients with low and high percentage of unmatched units (Fig. 3a, b, respectively).

### Distribution of ventilation and perfusion

**Fig. 4:**
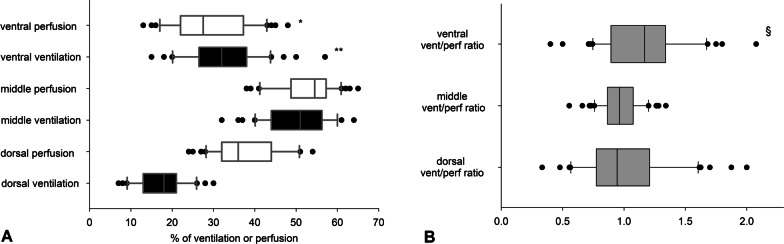
Regional distribution of ventilation, perfusion and ventilation/perfusion ratio. *p < 0.05 versus dorsal perfusion; **p < 0.05 versus dorsal ventilation; §*p* < 0.05 versus dorsal ventilation/perfusion ratio

**Fig. 5:**
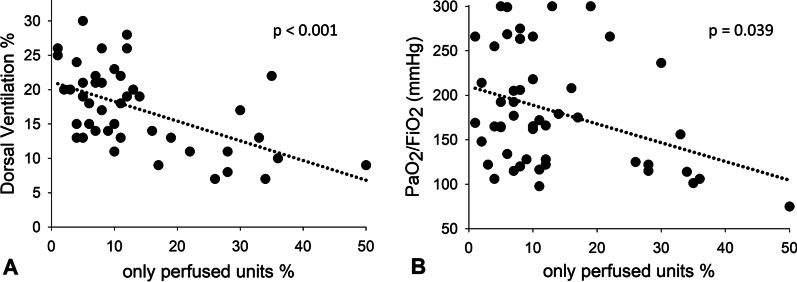
Correlation between percentage of only perfused units and dorsal ventilation and between percentage of only perfused unit and PaO_2_/FiO_2_. Higher percentage of only perfused units were correlated with lower dorsal ventilation (*r* =  − 0.486, *p* < 0.001) and with lower PaO_2_/FiO_2_ ratio (*r* =  − 0.293, *p* = 0.039)

In the whole population, the percentage of ventilation to the ventral region of the lung was higher than the percentage of ventilation to the dorsal region (ventral ventilation 32 [27–38]% vs. dorsal ventilation 18 [13–21] %, *p* < 0.001), while the opposite was true for perfusion (ventral perfusion 28 [22–38] % vs. dorsal perfusion 36 [32–44]%, *p* < 0.001) (Fig. 4a). As a result, relative value of ventilation/perfusion ratio was higher in ventral compared to dorsal region (1.2 [0.9–13] vs. 0.9 [0.8–1.2], *p* = 0.016) (Fig. 4b). Higher percentage of only perfused units were correlated with lower dorsal ventilation (*r* =  − 0.486, *p* < 0.001) (Fig. 5a).

A weak negative correlation was also found between percentage of only perfused units and PaO_2_/FiO_2_ ratio (*r* =  − 0.293, *p* = 0.039), suggesting that loss of dorsal ventilation contributes to the impairment of oxygenation due to intrapulmonary shunt (Fig. 5b).

### PEEP effect on ventilation/perfusion matching

We explored the correlation between PEEP and EIT-derived indexes of ventilation/perfusion matching in subgroups of patients with different ARDS characteristics.

**Fig. 6:**
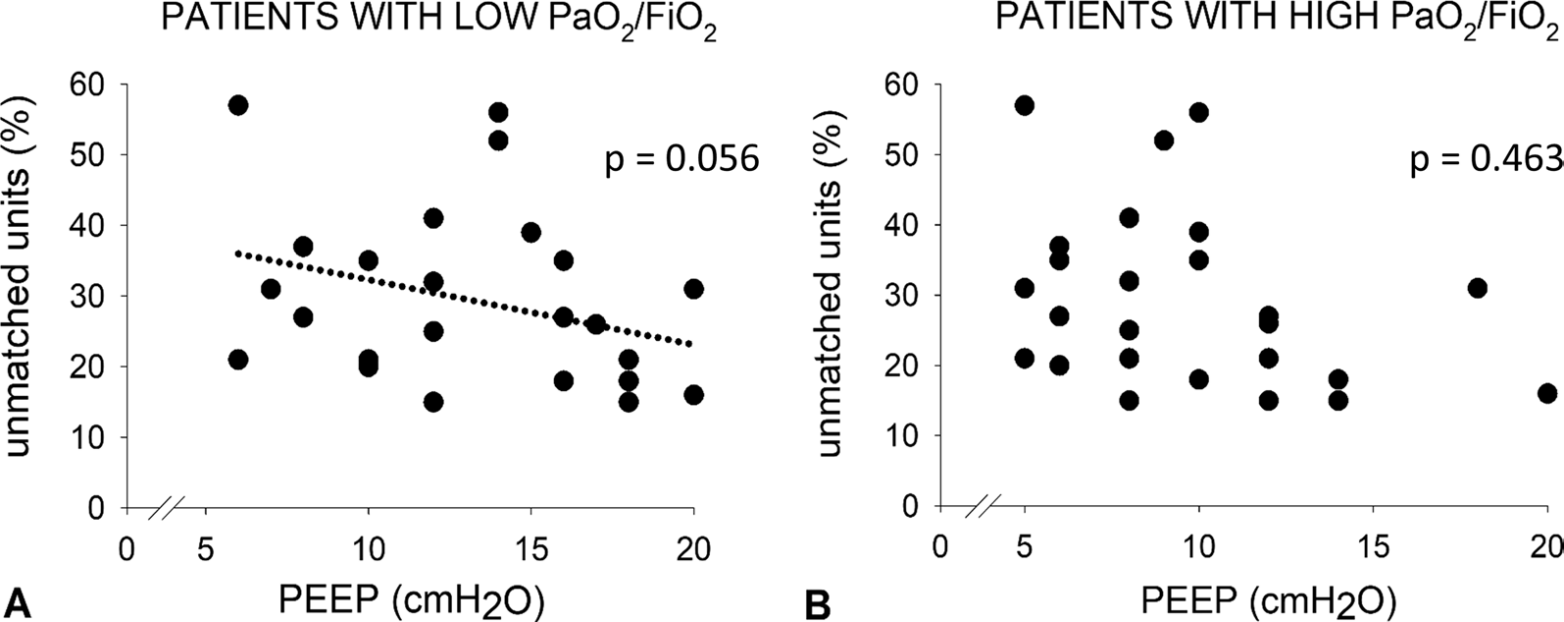
Correlation between PEEP and percentage of unmatched units in patients with low and high PaO_2_/FiO_2_. Higher PEEP values tend to correlate with lower percentage of unmatched units in the more severe subgroup with lower PaO_2_/FiO_2_ ratio (*r* =  − 0.387, *p* = 0.056), but not in the subgroup with higher PaO_2_/FiO_2_ ratio (*r* = 0.152, *p* = 0.465). The two subgroups were defined based on the median value of PaO_2_/FiO_2_ in the study population (lower or equal to 165 mmHg versus higher than 165 mmHg)

Higher PEEP was correlated with lower percentage of unmatched units in the more severe subgroup with PaO_2_/FiO_2_ ratio lower than the median value of 168 mmHg (*r* =  − 0.387, *p* = 0.056), but not in the subgroup with higher PaO_2_/FiO_2_ ratio (*r* = 0.152, *p* = 0.465) (Fig. 6).

**Fig. 7:**
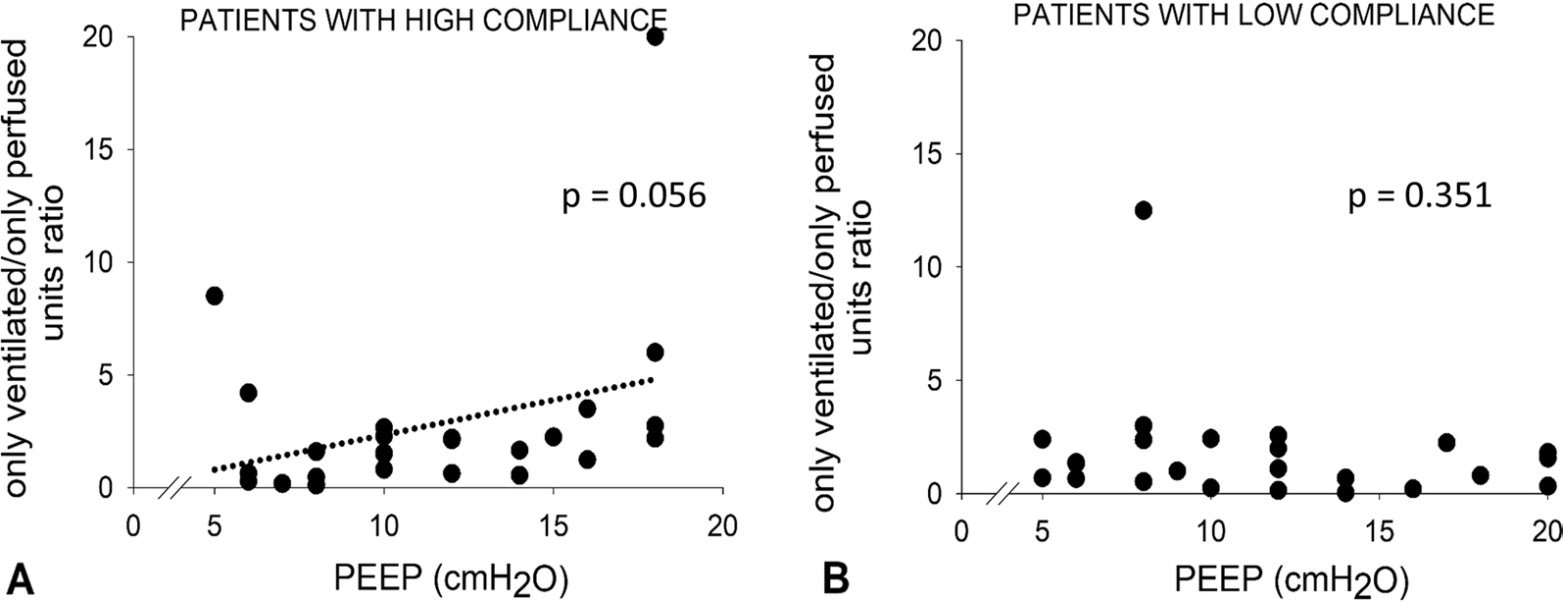
Correlation between PEEP and only ventilated/only perfused units in patients with low and high compliance. Higher PEEP was associated with higher only ventilated/only perfused units ratio) in the subgroup of patients with higher compliance (*r* = 0.386, *p* = 0.056), but not in the subgroup with lower compliance (*r* =  − 0.193, *p* = 0.351). The two subgroups were defined based on the median value of compliance of the respiratory system in the study population (lower or equal to 41 ml/cmH_2_O versus higher than 41 ml/cmH_2_O)

Higher PEEP was associated with higher only ventilated/only perfused units ratio in the subgroup of patients with compliance higher than the median value of 41 ml/cmH_2_O (*r* = 0.386, *p* = 0.056), but not in the subgroup with lower compliance (*r* =  − 0.193, *p* = 0.351) (Fig. 7).

## Discussion

We performed EIT-based measurements of the relative distribution of ventilation and perfusion and of the percentage of lung units with unmatched ventilation and unmatched perfusion in 50 patients with ARDS. The study has three main findings. First, the percentage of unmatched lung units (i.e., only perfused units plus only ventilated units) is an independent predictor of mortality in ARDS patients. Second, EIT allows bedside evaluation of the relative distribution of regional ventilation and perfusion within the ARDS lungs, which shows higher relative ventilation/perfusion ratio in ventral regions compared to dorsal regions. Loss of dorsal ventilation correlates with the percentage of unmatched units and with the impairment of oxygenation. Third, we explored the correlation between PEEP and ventilation/perfusion matching in subgroups of patients with low versus high PaO_2_/FiO_2_ and low versus high compliance.

Simple bedside methods for monitoring ventilation/perfusion matching are lacking in clinical practice. EIT-derived measurements of ventilation and perfusion integrate functional and anatomical information. In EIT images, only perfused units and only ventilated units are expressed as percentages of total lung units. This is in contrast with other methods to calculate dead space [27, 28] and shunt [29] which are “traditionally” defined as percentages of minute ventilation and cardiac output, respectively. Quantification of only ventilated units by EIT might be a more specific index of perfusion defects, because it is not affected by anatomic dead space nor by regions with high V/Q ratio [30, 31]. Likewise, measurement of only perfused units within the lung might disclose the “true” uncoupled perfusion due to loss of aeration, without including regions with low V/Q ratio [32]. Our finding on the predictive value of unmatched units (which are the sum of only ventilated plus only perfused units) for ARDS mortality may indicate the specificity of these EIT-based measures in assessing ARDS severity, and in the future, they may become a mean to evaluate effectiveness of personalized interventions. The ratio of only ventilated/only perfused units is another composite index of ventilation/perfusion imbalance provided by EIT. Although the same ratio might result from different absolute values of only ventilated and only perfused units, its value provides a simple measurement of the prevalent mechanism of gas exchange impairment, i.e., of the imbalance of V/Q mismatch towards dead space (higher values) or shunt (lower values). In fact, we found higher values of this index in ARDS due to COVID-19 [20] compared to this group of ARDS of other etiologies, supporting the hypothesis that, on average, the relative contribution of altered perfusion versus altered ventilation is opposite between the two types of ARDS.

EIT-based assessment of the regional distribution of ventilation and perfusion confirmed that relative ventilation/perfusion ratio is higher in the ventral compared to dorsal regions. Loss of ventilation of dorsal regions due to increased lung weight determines hypoxemia in ARDS when perfusion is preserved [33, 34]. However, the weak correlation between percentage of only perfused units and PaO_2_/FiO_2_ suggests that loss of ventilation is only one of the mechanisms for oxygenation impairment. Regions with low ventilation/perfusion ratio likely contribute, too [35], but they are not currently detected by EIT. Moreover, redistribution of lung perfusion due to reversal of hypoxic vasoconstriction [35, 36] might alter this correlation in patients undergoing ECMO.

The exploratory analysis on the physiologic effects of PEEP suggest that higher PEEP might be beneficial in patients with more severe hypoxia and detrimental in patients with higher compliance. Previous studies have demonstrated wide variability in the effect of PEEP on ventilation/perfusion matching [37–39]. A recent study using EIT showed that increasing PEEP improves matching when associated with a positive balance between recruitment and overdistension [21]. Lower percentage of unmatched units at higher PEEP suggests that this favorable balance mostly occurs in the subgroup with lower PaO_2_/FiO_2_, likely because true shunt assessed by only perfused units is the main mechanisms of oxygenation impairment at high FiO_2_ [32]. The lack of correlation between PEEP and the percentage of unmatched units in the subgroup with less severe hypoxia might be due to a lack of recruitment and/or to diversion of perfusion from more aerated to less aerated regions at higher PEEP [40, 41].

On the other hand, increased ratio of only ventilated/only perfused units at higher PEEP suggests that overdistension might prevail in patients with higher compliance. This confirms our previous observations in patients with ARDS due to COVID-19 [20]. In contrast with our findings, overdistension was associated with decreased percentage of only ventilated units in another study [24]. The effect of high PEEP on hemodynamics might explain these differences, as decreased ventral perfusion might prevent the increase in only ventilated units when cardiac output is decreased by PEEP [24]. In summary, these results confirm the wide variability in PEEP response and stress the need for a bedside method to monitor changes in ventilation/perfusion matching. Prone positioning might be a more effective and safer approach to revert dorsal loss of ventilation [42] and improve homogeneity of ventilation/perfusion matching [24].

This study has limitations. First, EIT measurements were performed under clinical rather than standardized ventilation settings and patients undergoing ECMO were included. However, this increases clinical translation of our findings. Second, EIT does not provide images of the whole lung, but it does provide a validated projection of a three dimensional distribution of ventilation and perfusion along cranial–caudal direction on a two-dimensional axial plane [16, 43]. We did not compare EIT-derived indexes of ventilation/perfusion matching with “classic” measurements of shunt and dead space. The aim of this clinical work was to explore the prognostic value of these indexes and not to perform a validation study. Moreover, further development of the EIT analysis of V/Q matching may be needed before comparing to these classic values: indeed, units with low and high V/Q ratio plays an important role but aren’t currently measured by EIT. Third, only ventilated and only perfused units were defined according to arbitrary thresholds representing a valid working compromise. As first exploratory analysis, we did not explore the entire spectrum of V/Q imbalances that occur within the lung. Fourth, since levels were not randomized nor standardized, results on the correlations between PEEP and VQ matching should be considered as exploratory. Moreover, we only assessed the effect of PEEP on overall unmatched units, while analysis of the regional effects of PEEP on ventilation and perfusion in dorsal and ventral regions might be more informative. Further development of the analysis could aim at obtaining regional data from EIT-based measurements of ventilation and perfusion. Fifth, accurate removal of the cardiac region is relevant to analyze ventilation/perfusion limited to lung pixels, avoiding to include some ventral lung perfusion which is actually cardiac perfusion. The combination of pulsatility and indicator dilution is a conservative approach which should minimize this error.

## Conclusions

Electrical impedance tomography allows bedside noninvasive assessment of only ventilated and only perfused units in mechanically ventilated patients with ARDS. The percentage of unmatched units is an independent predictor of mortality. The effect of PEEP on unmatched units and on the ratio between only ventilated and only perfused units might differ in ARDS subphenotypes based on oxygenation and compliance. Bedside assessment of ventilation/perfusion matching by EIT might enhance the characterization of ARDS, identify patients at higher risk of death and, in turn, guide personalized treatment.

## Data Availability

The datasets used and/or analyzed during the current study are available from the corresponding author on reasonable request.

## Abbreviations

ARDS: Acute respiratory distress syndrome
VILI: Ventilation-induced lung injury
EIT: Electrical impedance tomography
PEEP: Positive end expiratory pressure
ICU: Intensive Care Unit
Vt: Tidal volume
RR: Respiratory rate
FiO_2_: Fraction of inspired oxygen
SAPS II: Simplified Acute Physiology Score II
VFDs: Ventilator-free days
V/Q: Ventilation/perfusion
ROC: Receiver operating characteristics
IQR: Interquartile range
ECMO: Extracorporeal membrane oxygenation

## Declarations

Dräger company had no role in the conception and design of the study and in the enrolment of the patients. Drager supported the study by collaboration on the development of the software for perfusion data analysis and by assistance for explanation of technical aspects. Dräger had no access to demographic and clinical patient data. Dräger had no role in the decision to publish the results. However, all authors collaborated to drafting the manuscript, reviewed it for important intellectual content and agreed on the final version.
